# HIV, Nephrotoxic Medications, and Chronic Kidney Disease: Prevalence, Risk Factors, and Mediation Analyses Among People With and Without HIV Enrolled in the Multicenter AIDS Cohort Study (MACS) / Women’s Interagency HIV Study (WIHS) Combined Cohort Study

**DOI:** 10.1101/2025.10.30.25339018

**Published:** 2025-11-06

**Authors:** Yue Pan, Dominique Musselman, Zain Mithani, Weiqun Tong, Joseph Margolick, Frank J. Palella, Matthew J. Mimiaga, Kaitlin Bodnar, Deborah Konkle-Parker, Gina Wingood, Daniel Westreich, Eric Seaberg, Signe Lauren, Mardge Cohen, Michelle M. Estrella, Amanda Spence, Tracey Wilson, Michael Ross, Dan Feaster, Maria L. Alcaide, Deborah L. Jones

**Affiliations:** 1. Department of Public Health Sciences, University of Miami; 2. Department of Psychiatry and Behavioral Sciences, University of Miami; 3. Division of Infectious Diseases, Department of Medicine, University of Miami Miller School of Medicine; 4. Department of Epidemiology, Johns Hopkins Bloomberg School of Public Health; 5. Department of Molecular Microbiology and Immunology, Johns Hopkins Bloomberg School of Public Health; 6. Department of Medicine, Division of Infectious Diseases, Northwestern University Feinberg School of Medicine; 7. Department of Epidemiology, UCLA Fielding School of Public Health and Department of Psychiatry and Biobehavioral Sciences, UCLA Geffen School of Medicine, Los Angeles, CA, USA; 8. Department of Medicine, Division of Infectious Diseases, University of Pittsburgh; 9. Schools of Nursing, Medicine and Population Health, University of Mississippi Medical Center; 10. Department of Sociomedical Sciences, Mailman School of Public Health, Columbia University; 11. Department of Epidemiology, Gillings School of Global Public Health, University of North Carolina at Chapel Hill; 12. Department of Medicine, Rush University Medical Center; Cook County Health & Hospitals System; 13. Division of Nephrology, Department of Medicine, University of California, San Francisco; San Francisco VA Health Care System; 14. Division of Infectious Diseases, Department of Medicine, Georgetown University; 15. Department of Community Health Sciences, School of Public Health, SUNY Downstate Health Sciences University; 16. Department of Medicine (Nephrology), Albert Einstein College of Medicine; 17. Department of Medicine, OB/GYN, and Public Health Sciences. University of Miami Miller School of Medicine

## Abstract

**Background:**

Chronic kidney disease (CKD) affects over 37 million adults in the United States, and people living with HIV (PLWH) are at greater risk for progression to end-stage kidney disease. Although both conditions are common among PLWH, the potential pathways through which depression and use of medications with nephrotoxic potential may influence CKD development remain underexplored. We evaluated the relationships of depression and nephrotoxic medication use with CKD prevalence among PLWH, and investigated the potential mediating effects of these factors on the pathway to CKD among PLWH.

**Methods:**

We analyzed longitudinal data from the Multicenter AIDS Cohort Study (MACS)/Women’s Interagency HIV Study (WIHS) Combined Cohort Study (MWCCS), collected between October 2018 and September 2021, to assess the prevalence of kidney dysfunction (eGFR <60 mL/min/1.73 m^2^) and its association with HIV serostatus. Generalized Estimating Equations (GEE) with a Poisson distribution and log link were used to estimate relative risks (RR) for CKD associated with HIV, depression, and other covariates. Counterfactual-based causal mediation analysis was conducted to assess whether depressive symptoms or nephrotoxic medication use partially explained the observed association between HIV and CKD.

**Results:**

Among 2,530 participants [1,622 PLWH and 908 people living without HIV (PLWoH)], CKD prevalence was higher in PLWH (18.1%) compared to PLWoH (9.7%). GEE analysis revealed that HIV serostatus was significantly associated with an increased risk of CKD (RR = 1.37, 95% CI: 1.28–1.48, p < 0.0001). Other significant factors included age (RR = 1.03, 95% CI: 1.03–1.03, p < 0.0001), non-Hispanic Black (RR = 1.19, 95% CI: 1.11–1.27, p < 0.0001) compared to non-Hispanic White, diabetes (RR = 1.26, 95% CI: 1.17–1.35, p < 0.0001), and higher income (RR = 0.99, 95% CI: 0.98–1.00, p = 0.005). Mediation analysis indicated that nephrotoxic medication use, accounted for a small but significant proportion of the HIV- CKD association (indirect effect OR = 1.02, 95% CI: 1.00–1.03, p = 0.02).

**Conclusions:**

While it is well established that PLWH have a higher prevalence of CKD compared to PLWoH, our findings suggest that nephrotoxic medication use may modestly amplify this risk. Although most of the risk appears to be attributable to the direct effects of HIV, these medications represent a modifiable contributor. PLWH receiving such treatments may benefit from closer kidney function monitoring. Future research should investigate additional psychosocial and behavioral contributors to CKD among PLWH, including depression.

## Introduction

People living with HIV (PLWH) enjoy markedly extended survival due to the routine use of virally suppressive antiretroviral therapy. However, PLWH remain at elevated risk for kidney-related complications, including a higher incidence of acute kidney injury and a greater burden of both general (e.g., hypertension and diabetes) and HIV-specific risk factors for chronic kidney disease (CKD). The prevalence of HIV-associated chronic kidney disease (CKD) varies widely by region, patient characteristics, reporting methods, and CKD definition. In North America and Europe, using a definition of eGFR <60 mL/min/1.73 m^2^ or proteinuria, prevalence may reach 15% or higher [1].

The development of CKD in PLWH is often multifactorial. It may result from HIV-related immune dysfunction (e.g., prolonged immunosuppression, high viral load), genetic predisposition, and the use of certain antiretroviral medications [2]. Compared with the general population, PLWH experience a 2- to 20-fold greater risk of developing end-stage kidney disease [1, 3], with a disproportionately higher risk borne by Black individuals [4]. Even more fundamentally, HIV is directly kidney-tropic: in the pre-ART era, HIV-associated nephropathy (HIVAN), characterized by rapid progression to renal failure and collapsing focal segmental glomerulosclerosis was a leading cause of death among Black PLWH [5]. The kidney, as one of HIV’s primary targets, remains a critical focus for renal prevention and therapeutic strategies.

Depression is also common amongst PLWH, occurring in about 28% of PLWH who are older than 50 years of age [6]. Two recent community-based studies highlighted the relationship between depression and CKD. The China Health and Retirement Longitudinal Study enrolled 4763 participants with eGFR≥60 ml/min per 1.73 m^2^ at baseline and discovered that in persons without kidney disease at baseline, those with depressive symptoms were more likely to show signs of rapid kidney function decline over a median follow-up of 4 years and suffered 39% greater chance of rapid kidney decline, independent of other major demographic, clinical, or psychosocial covariates [7]. The second study highlighted the synergistic impact of depression in persons in the United States (US) who had already developed CKD. The 2022 retrospective analysis of 24,412 participants within the National Health and Nutrition Examination Survey 2005–2014 had a mean follow-up of 5.8 years. Persons with CKD with comorbid depression exhibited over three and a half times the hazard ratio for all-cause and cardiovascular-related mortality, followed by persons with depression without CKD, then persons with CKD only [8]. However, neither study examined the potential role of nephrotoxic medications, nor did they evaluate these relationships in the context of HIV serostatus [9].

Nephrotoxic medications, including antidepressants and antipsychotics, are commonly prescribed to manage depression in PLWH, but some of these medications have known nephrotoxic properties that may exacerbate kidney dysfunction [10]. Nephrotoxic medications may play a mediating role in the relationship between HIV and CKD, either through their nephrotoxic effects or by influencing depression-related pathways that affect kidney function. As PLWH suffer increased rates of depression compared to PLWoH, and depression has been repeatedly associated with increased morbidity and mortality in persons with CKD [11, 12], evaluating the impact of depression and nephrotoxic medication usage on kidney function in PLWH is critical.

In this study, we aimed to evaluate the prevalence and incidence of CKD among PLWH compared to people living without HIV (PLWoH), and to examine whether depression and the use of nephrotoxic medications were independently associated with CKD. We leveraged data from the Multicenter AIDS Cohort Study (MACS)/Women’s Interagency HIV Study (WIHS) Combined Cohort Study (MWCCS), the largest and longest-running prospective cohort of individuals with and without HIV in the United States, to address these questions and inform targeted clinical strategies for kidney health in this population.

Given the high prevalence of depression among PLWH, its known associations with chronic illness outcomes, and the widespread use of nephrotoxic medications, many of which have nephrotoxic potential, it is critical to examine how these factors may contribute to CKD risk in this population. We hypothesized that depression would be associated with increased CKD risk among PLWH. Additionally, we conducted causal mediation analyses to determine whether depressive symptoms and the use of nephrotoxic medications served as mediators in the relationship between HIV serostatus and CKD, thereby identifying potentially modifiable factors along this pathway.

## Materials and methods

### Study Design and Population

The MWCCS is a prospective observational cohort study designed to study the impact of chronic health conditions that affect PLWH in the US. Details have been previously described [13]. For this longitudinal analysis, baseline data were provided by the MWCCS Data Coordinating Center on participants aged 30 and older at the 13 MWCCS sites. Data were derived from three consecutive visits, identified as baseline (Visit 101), follow-up 1 (Visit 102), and follow-up 2 (Visit 103), occurring between November 16, 2020, and March 30, 2023. Specifically, Visit 101 spanned from November 16, 2020, to September 30, 2021; Visit 102 occurred from October 4, 2021, to September 30, 2022; and Visit 103 extended from October 1, 2022, to March 30, 2023. At Visit 101, 2,530 participants were enrolled as new participants. At Visit 102, 1,030 new participants joined, while 1,958 participants from Visit 101 were retained. By Visit 103, 460 new participants enrolled, and 892 participants from Visit 102 continued their participation. Across all three visits, a total of 6,870 participant records were analyzed, including 4,020 new participants and 2,850 returning participants.

This study analyzed baseline demographic, clinical, and behavioral characteristics of participants stratified by HIV serostatus to evaluate the prevalence of CKD. The analysis was conducted from 1,622 PLWH and 908 PLWoH in MWCCS. Variables were categorized and constructed based on participant demographics, socioeconomic factors, clinical diagnoses, behavioral data, and laboratory measurements. The preliminary cross-sectional analyses utilized baseline data from study subjects at visit 101, conducted between October 2020 and September 2021, across all participating MWCCS sites. Demographic data include age, gender, race, and laboratory measures for eGFR calculated using the Chronic Kidney Disease Epidemiology Collaboration (CKD-EPI) equation. Additional covariates at baseline include HIV serostatus, diabetes mellitus, hypertension, cardiovascular disease, body mass index (BMI), and substance use (alcohol, tobacco, injection drug use (IDU), cocaine, opiates). Socioeconomic factors, including education, marital status, and annual household income, were also considered. Kidney disease was indexed based on self-reported history of kidney disease diagnosis by a healthcare provider (e.g., doctor, nurse).

### Assessments of Kidney Function, Depressive Symptoms, and Nephrotoxic Medication Use

CKD was defined as eGFR <60 ml/min per 1.73 m2 (using the CKD-EPI equation) for >3 months [14], consistent with the Kidney Disease: Improving Global Outcomes (KDIGO) 2012 clinical practice guidelines for the evaluation and management of CKD. In addition, kidney function was categorized based on estimated glomerular filtration rate (eGFR) into six stages to reflect progressive kidney damage and loss of function. The stages include Kidney Damage with Normal Kidney Function (eGFR ≥ 90 ml/min/1.73 m^2^), Kidney Damage with Mild Loss of Kidney Function (eGFR ≥ 60 and < 90 ml/min/1.73 m^2^), Mild to Moderate Loss of Kidney Function (eGFR ≥ 45 and < 60 ml/min/1.73 m^2^), Moderate to Severe Loss of Kidney Function (eGFR ≥ 30 and < 45 ml/min/1.73 m^2^), Severe Loss of Kidney Function (eGFR ≥ 15 and < 30 ml/min/1.73 m^2^), and Kidney Failure (eGFR < 15 ml/min/1.73 m^2^).

Depression was measured using the Center for Epidemiologic Studies Depression (CES-D) 20-item questionnaire [15]. A cut-off score of ≥16 was used to indicate elevated depressive symptoms, consistent with established screening thresholds applied in both men and women in large cohort studies, including the MACS and WIHS cohorts [16, 17].

Nephrotoxic medications were defined as medications with established nephrotoxic properties, based on expert review and retained for analysis in this study. These medications were classified based on their potential nephrotoxic effects and nephrotoxic properties, ensuring clinical relevance in assessing exposure. The medications were assessed at the time of the visit, capturing both current use and historical medication exposure for those who may have used them in the past [18]. Medications with nephrotoxic potential were identified using a modified Delphi method, which involved expert consensus across multiple rounds to systematically review and classify medications based on their likelihood of causing kidney toxicity [19]. (Full category list is provided in Supplement Table 1). This list reflects only medications with nephrotoxic properties relevant to CKD risk.

### Assessment of Covariates

Demographics include self-reported sex at birth, race/ethnicity, and age. Race/ethnicity categories included non-Hispanic white, non-Hispanic Black, non-Hispanic other, and Hispanic. As for socioeconomic indicators, education level (highest level completed) was categorized into: less than high school, completed high school, some college, completed a four-year college degree, and completed graduate school. Marital status was classified into legally/common-law married, living with a partner, widowed, divorced, separated, never married, and other. Employment status included categories of full-time, part-time, not employed, retired, student, and disability. Income was categorized into predefined annual household income brackets. Diabetes and hypertension status were binary indicators derived from medical diagnoses or medication use. Other clinical factors included BMI, categorized into standard weight categories (e.g., underweight, normal weight, overweight, obese). Smoking status was defined as currently smoking (yes/no). Alcohol use was categorized based on binge drinking episodes since the last visit. Drug use included self-reported use of marijuana, cocaine, heroin, or other illicit drugs, as well as injection drug use.

### Statistical Analyses

Descriptive statistics were utilized to summarize baseline demographic, clinical, and behavioral characteristics of the participants stratified by HIV serostatus. For continuous variables, means and standard deviations (SD) were calculated, along with medians and ranges. Comparisons between PLWH and PLWoH were assessed using analysis of variance (ANOVA) for continuous variables. For categorical variables, frequencies and percentages were reported, and chi-square tests were employed to evaluate differences between groups.

For the prevalence analysis, we used data from Visit 101 (baseline) only. CKD prevalence was calculated as the proportion of participants meeting the CKD definition (eGFR <60 mL/min/1.73 m^2^) within each subgroup of interest. Results were stratified by HIV serostatus (overall, PLWH, and PLWoH) and by demographic, clinical, and behavioral characteristics.

To examine associations between risk factors and CKD, generalized estimating equations (GEE) with a Poisson distribution and log link were used to estimate relative risks while accounting for within-subject correlation across multiple study visits. Separate univariate models were fitted for each covariate to avoid potential overadjustment and misinterpretation due to collider or mediator bias (i.e., Table 2 fallacy [20]). The analysis included both categorical and continuous covariates, and relative risks (RR) and 95% confidence intervals (CI) were computed for each predictor.

The GEE models incorporated an exchangeable working correlation matrix and accounted for repeated measurements clustered within individuals. Variables examined included HIV serostatus (PLWH vs. PLWoH), age, sex, race/ethnicity, marital status, education level, income, diabetes, hypertension, depression, nephrotoxic medication use, smoking, alcohol use, other substance use, and BMI. For categorical variables with more than two levels (e.g., education, marital status, race/ethnicity), comparisons were made relative to the reference group. Statistical significance was determined using Wald tests, and model fit was evaluated using empirical standard errors and robust variance estimates.

In addition, we performed counterfactual mediation analysis to quantify the pathway-specific effects of HIV on CKD through depression and nephrotoxic medication use. Using a binomial model with a logit link, we estimated the *total effect (TE)*, the *natural direct effect (NDE)*, and the *natural indirect effect (NIE)* on the odds-ratio scale, adjusting for sex, race, marital status, diabetes, hypertension, smoking, and substance use. The NDE represents the effect of HIV on CKD if the mediator were set to the level it would naturally take under the reference (PLWoH) exposure; the NIE represents the portion operating through the mediator. We allowed for exposure mediator interaction. The primary goal was to determine the extent to which depression or nephrotoxic medication use mediated the pathway from HIV to CKD, while accounting for potential interaction between the exposure and the mediator. Bootstrap resampling with 1,000 iterations was used to derive robust 95% confidence intervals. A two-sided p-value of <0.05 was considered statistically significant.

## Results

### Study Participants and Baseline Characteristics

The study included 1,622 PLWH and 908 PLWoH participants (Table 1). PLWH had a slightly younger mean age (54.7 years) compared to PLWoH (56.0 years, p=0.002). A higher proportion of PLWH were female (73.4%) compared to PLWoH (57.5%, p<0.001). The majority of participants were non-Hispanic Black individuals (66.0% of PLWH and 54.1% of PLWoH). Socioeconomic indicators revealed disparities in education and income levels, with PLWH more likely to have lower educational attainment and income levels than PLWoH (p<0.001).

Significant differences were observed in the prevalence of CKD, with 18.1% of PLWH affected compared to 9.7% of PLWoH participants (p<0.001). Additionally, measures of kidney function indicated worse outcomes among the PLWH, with fewer participants retaining normal kidney function (31.3% vs. 46.3%, p<0.001).

### Prevalence of CKD overall and stratified by HIV serostatus

Table 2 presents CKD prevalence across demographic, clinical, and behavioral characteristics, stratified by HIV serostatus. Higher CKD prevalence was observed among females, individuals with lower educational attainment, and persons identifying as non-Hispanic Black or non-Hispanic Other. Among marital categories, widowed and “other” individuals exhibited elevated CKD rates. Participants who were retired or had a disability showed higher CKD prevalence compared to those employed full-time. Behavioral factors such as current smoking, binge drinking, and illicit drug use were associated with higher CKD prevalence, particularly among PLWH.

Depression showed a nuanced association with CKD. Among individuals without depression, CKD prevalence was 15.3%, higher in PLWH (18.3%) than PLWoH (10%). Among those with depression, overall CKD prevalence was 13.9%, also higher in PLWH (17.1%) than PLWoH (8.5%). At Visit 101, 1,342 participants (53%) were classified as using nephrotoxic medications (Table 3). Nephrotoxic medication use was 51.9% in PLWoH and 53.7% in PLWH.

### Generalized Estimating Equations (GEE) Analysis of Kidney Function Decline

Table 4 presents the relationship between HIV status, depression, and chronic kidney disease (CKD). PLWH had a significantly higher risk of kidney function decline compared to PLWoH (RR = 1.37, 95% CI: 1.28–1.48, p < 0.001). Age was also positively associated with poorer kidney function, with each additional year of age associated with a 3% increase in CKD risk (RR = 1.03 per year, 95% CI: 1.03–1.03, p < 0.001).

Among racial/ethnic groups, non-Hispanic Black individuals had a significantly higher risk (RR = 1.19, 95% CI: 1.11–1.27, p < 0.001), and Hispanic individuals had more than twice the risk compared to non-Hispanic White individuals (RR = 2.03, 95% CI: 1.86–2.21, p < 0.001). Additionally, non-Hispanic Other individuals also showed an elevated risk (RR = 2.64, 95% CI: 2.31–3.02, p < 0.001). Sex was also significantly associated, with females having higher CKD risk compared to males (RR = 1.16, 95% CI: 1.08–1.23, p < 0.001). Higher income was associated with lower CKD risk (RR = 0.99, 95% CI: 0.98–1.00, p = 0.005). Among chronic conditions, diabetes was significantly associated with increased risk of CKD (RR = 1.26, 95% CI: 1.17–1.35, p < 0.001), while hypertension was not statistically significant (RR = 1.06, 95% CI: 0.97–1.15, p = 0.204).

**Figure 1.**
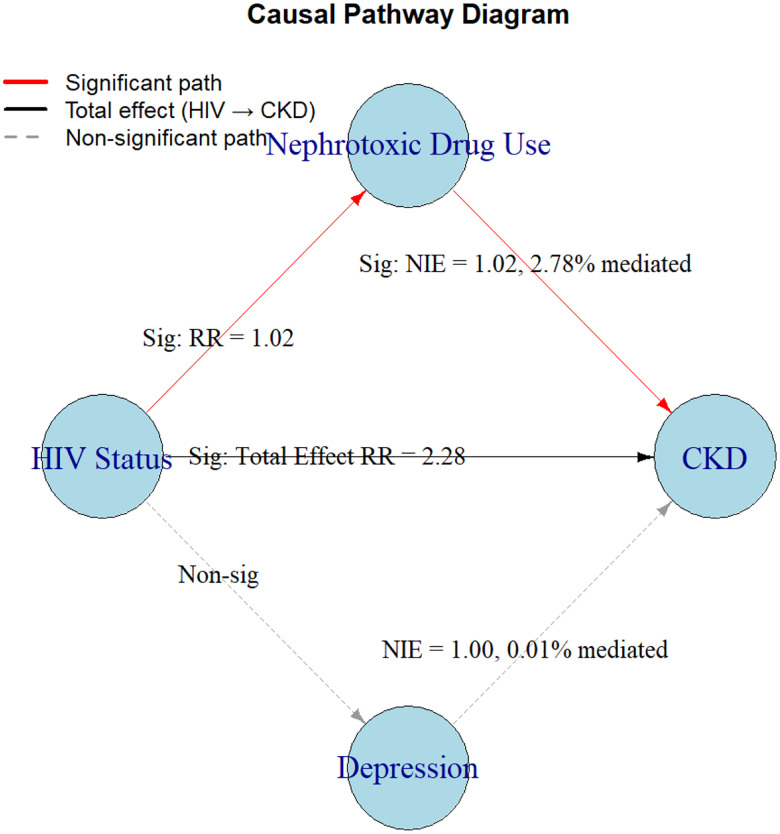
Causal Pathway Diagram

Depression or Nephrotoxic medication use mediates the relationship between HIV Status and CKD. We conducted a mediation analysis to evaluate whether depression and nephrotoxic medication use mediated the relationship between HIV status and CKD. The total OR of CKD associated with HIV status was 2.28 (95% CI: 1.88–2.77, p < 0.0001; Fig 1), indicating a strong association between HIV and CKD. The natural direct effect (NDE), i.e., portion of the HIV–CKD association not operating through nephrotoxic medication use, was similar in magnitude (OR = 2.25, 95% CI: 1.84–2.73, p < 0.0001).

The natural indirect effect (NIE) through nephrotoxic drug use was modest but statistically significant (OR = 1.02, 95% CI: 1.00–1.03, p = 0.019), accounting for ~2.78% of the total effect. Most of the association was explained by the direct pathway, with a small yet meaningful portion mediated through nephrotoxic drug exposure. Importantly, the interaction between HIV and nephrotoxic drug use was statistically significant (17.83%, p = 0.0002), suggesting that nephrotoxic drug exposure further increased CKD risk among people living with HIV.

## Discussion

While previous studies have suggested a strong link between depression and CKD progression, our analysis did not find a direct relationship. This lack of association may be due to effective treatment and management of depression in this cohort, which could have mitigated the effects on kidney function. In contrast, our mediation analysis revealed that nephrotoxic medication use played a modest but significant role in mediating the relationship between HIV and CKD. These agents have well-documented nephrotoxic effects that could exacerbate kidney damage. Thus, the mediating role of nephrotoxic medications in the HIV - CKD pathway is likely driven by their nephrotoxicity. Importantly, the interaction between HIV and nephrotoxic drug use was statistically significant, suggesting that nephrotoxic drug exposure amplifies CKD risk in PLWH. These findings highlight the need for clinicians to routinely assess kidney function when prescribing or managing nephrotoxic medications in PLWH and to weigh renal risks alongside clinical benefits as part of personalized HIV care.

Following this key finding, we examined the broader relationships between HIV serostatus, depression, nephrotoxic medication use, and CKD. First, consistent with prior research [1], we observed a high prevalence of CKD, and a significantly higher prevalence of CKD in PLWH (18.1%) compared to PLWoH (9.7%). This disparity remained significant after adjusting for demographic, clinical, and behavioral covariates. Furthermore, the longitudinal analysis using GEE confirmed that HIV serostatus was a strong predictor of kidney function decline. Our findings are congruent with prior studies documenting that kidney disease in PLWH is a common and serious complication, with reports documenting kidney disease as the fourth leading cause of death in patients with AIDS prior to the use of effective ART and among individuals not using ART. Although HIV-associated nephropathy (HIVAN), especially in individuals carrying APOL1 risk alleles, was once the most common form of kidney injury in PLWH, its incidence has declined dramatically in the current ART era [21]. Today, other forms of CKD, such as diabetic kidney disease and hypertensive nephrosclerosis, are more frequently observed [14, 21–25]. HIV can also lead to renal injury through mechanisms such as HIV-associated thrombotic microangiopathy (TMA), acute interstitial nephritis, immune complex-mediated glomerular disease, and sepsis-related acute tubular necrosis (ATN) due to immunosuppression [26].

Fortunately, compared to people without HIV, kidney dysfunction in PLWH has declined with the use of modern ART and may now progress at a similar or slower rate compared to those without HIV. However, many medications used to treat HIV and the complications of HIV can result in renal injury. Protease inhibitors, such as atazanavir, have been associated with crystal-induced obstructive acute kidney injury (AKI) [27]. Tenofovir (a nucleoside reverse transcriptase inhibitors) use has been implicated in tubulopathies causing Fanconi syndrome and nephrogenic diabetes insipidus. Moreover, the presence of traditional risk factors such as hypertension, diabetes, and older age, remained critical determinants of CKD progression across both groups in our study.

Of note, individuals in our cohort with depression had a slightly lower prevalence of CKD compared to those without depression among PLWH. However, among PLWoH, CKD prevalence was also lower in those with depression compared to those without. These findings contrast with prior literature that suggests a strong link between depression and CKD progression [28–31], that is, there is a higher prevalence of depression as CKD worsens. One possible explanation for this discrepancy is that the MWCCS cohort includes individuals with more stable access to care and effective management of both HIV and mental health conditions. This may have attenuated the impact of depression on CKD progression. Studies such as those by Capuron et al. and Lustman et al. have suggested that while somatic depressive symptoms (e.g., fatigue, appetite loss) are more prevalent in conditions like diabetes, cognitive symptoms (e.g., sadness, hopelessness) may be more prominent in populations of persons with [12, 32]. In contrast, another group showed that in persons with cancer, the most optimal diagnostic tool for the identification of depression contained *both* somatic and cognitive symptoms (late insomnia, agitation, psychic anxiety, diurnal mood variation, depressed mood, and decreased libido) of the Hamilton Depression Rating Scale [33]. However, the lack of a significant mediating effect of depression on CKD progression in our analysis suggests that, although depression is highly prevalent among individuals with CKD, it may not independently contribute to disease progression. One possible explanation is that participants with elevated depressive symptoms may have benefited from better access to supportive services or clinical follow-up that attenuated CKD risk. Nevertheless, because depression in this study was defined solely based on CES-D symptom scores rather than clinical diagnosis or treatment history, we cannot determine whether participants were receiving appropriate or effective depression management. The potential modifying effect of depression on CKD risk, particularly among individuals with HIV, remains unclear and warrants further investigation in future studies that incorporate both symptom-based and treatment-based assessments.

Despite the strong association between HIV and CKD, the mediation analysis indicated that nephrotoxic medication use accounted for a small but significant proportion of this relationship. These findings align with prior studies suggesting that certain nephrotoxic drugs, including commonly used antiretrovirals and other agents, may contribute to kidney injury over time [30, 34–37].

### Strengths and Limitations

Our study has several strengths. A key strength is our use of mediation analysis to disentangle the direct and indirect effects of HIV on CKD, thereby allowing us to gain new insights into the role of nephrotoxic medication exposure in this association among PLWH. Additionally, the longitudinal design allowed us to assess CKD progression over time and explore differences in HIV serostatus, depression, and medication use. The MWCCS cohort also represents a deeply phenotyped, diverse population.

However, there are limitations to consider. The observational nature of our study precludes causal inferences, and unmeasured confounding variables, such as levels of inflammation markers (e.g., C-reactive protein) and alternative kidney function measures (e.g., cystatin C), were not available in our dataset. Inflammation, medication adherence, and healthcare access may influence both depression and CKD risk. These markers may have provided a more accurate estimation of kidney function, particularly in individuals with low muscle mass [38]. Medication data were limited to nephrotoxic drug exposures captured in the MWCCS cohort. As a result, while we were able to quantify the mediating effect of nephrotoxic medications overall, we could not evaluate the impact of non-nephrotoxic psychiatric medications because they were not captured in the coded data during the study window. This restricts the generalizability of our findings and should be interpreted accordingly.

## Conclusion

In conclusion, our study reaffirms the strong association between HIV and CKD, even among PLWH successfully treated with ART, with most of the risk explained by direct effects rather than mediation through depression. While depression was highly prevalent among persons with CKD, it was not independently associated with CKD in multivariable models. In contrast, nephrotoxic medication use played a modest but significant mediating role, suggesting that the contribution of medications to CKD risk in PLWH is largely driven by their renal toxicity. These findings highlight the need for careful renal monitoring in patients prescribed medications with nephrotoxic potential. Future research should further explore the biological mechanisms linking HIV, depression, and kidney disease, with particular attention to both medication-related toxicity and systemic inflammatory pathways.

## Supplementary Material

Supplement 1

## Figures and Tables

**Table 1:** Baseline Demographic, Clinical, and Behavioral Characteristics of Participants by HIV Status

	PLWH (N=1622)	PLWoH (N=908)	P-value
**Chronic Kidney Disease**			<0.001
No	1328 (81.9%)	820 (90.3%)	
Yes	294 (18.1%)	88 (9.7%)	
**Participant’s Age at Visit**			0.002
Mean (SD)	54.7 (9.81)	56.0 (11.7)	
Median [Min, Max]	56.0 [29.0, 85.0]	57.0 [30.0, 84.0]	
**Sex at Birth**			<0.001
Female	1190 (73.4%)	522 (57.5%)	
Male	432 (26.6%)	386 (42.5%)	
**Race/Ethnicity**			<0.001
Non-Hispanic White	297 (18.3%)	275 (30.3%)	
Non-Hispanic Black	1070 (66.0%)	491 (54.1%)	
Non-Hispanic Other	62 (3.8%)	32 (3.5%)	
Hispanic	193 (11.9%)	110 (12.1%)	
**Education**			<0.001
Less than high school	428 (26.4%)	195 (21.5%)	
Completed high school	452 (27.9%)	190 (21.0%)	
Some college	438 (27.0%)	228 (25.2%)	
Completed four-year college degree	162 (10.0%)	120 (13.2%)	
Attended/Completed graduate school	142 (8.8%)	173 (19.1%)	
**Marital Status**			<0.001
Legally/Common-law Married	322 (20.3%)	223 (25.2%)	
Not Married but Living with Partner	116 (7.3%)	102 (11.5%)	
Widowed	134 (8.4%)	45 (5.1%)	
Divorced	201 (12.7%)	90 (10.2%)	
Separated	83 (5.2%)	54 (6.1%)	
Never Married	626 (39.5%)	321 (36.2%)	
Other	104 (6.6%)	51 (5.8%)	
**Employment Status**			<0.001
Disability	560 (35.4%)	189 (21.4%)	
Employed Full-Time	442 (27.9%)	274 (31.0%)	
Employed Part-Time	156 (9.8%)	86 (9.7%)	
Not Employed	268 (16.9%)	170 (19.2%)	
Retired	149 (9.4%)	160 (18.1%)	
Student	9 (0.6%)	6 (0.7%)	
**Income**			<0.001
$6000 or less	109 (7.1%)	73 (8.6%)	
$6001–$12,000	470 (30.6%)	173 (20.4%)	
$12,001–$18,000	214 (13.9%)	96 (11.3%)	
$18,001–$24,000	139 (9.1%)	59 (6.9%)	
$24,001–$30,000	110 (7.2%)	56 (6.6%)	
$30,001–$36,000	85 (5.5%)	55 (6.5%)	
$36,001–$75,000	233 (15.2%)	161 (18.9%)	
$75,001–$100,000	57 (3.7%)	55 (6.5%)	
$100,001–$150,000	64 (4.2%)	62 (7.3%)	
$150,001–$200,000	30 (2.0%)	26 (3.1%)	
More than $200,000	24 (1.6%)	34 (4.0%)	
**Race free estimated Glomerular Filtration Rate (ml/min/1.73 m^2)**			<0.001
Mean (SD)	78.6 (21.1)	86.2 (19.5)	
Median [Min, Max]	79.7 [5.10, 127]	88.5 [4.93, 135]	
**Kidney Function (eGFR)**			<0.001
Normal Kidney Function (≥90)	507 (31.3%)	420 (46.3%)	
Mild Loss of Kidney Function (≥60 and <90)	821 (50.6%)	400 (44.1%)	
Mild to Moderate Loss of Kidney Function (≥45 and <60)	202 (12.5%)	56 (6.2%)	
Moderate to Severe Loss of Kidney Function (≥30 and <45)	54 (3.3%)	21 (2.3%)	
Severe Loss of Kidney Function (≥15 and <30)	23 (1.4%)	8 (0.9%)	
Kidney Failure (<15)	15 (0.9%)	3 (0.3%)	
**Body Mass Index (kg/m^2^)**			0.702
Mean (SD)	31.7 (8.74)	31.6 (8.26)	
Median [Min, Max]	30.0 [12.9, 75.6]	30.0 [14.1, 67.8]	
**Has Diabetes**			0.253
No	1224 (75.6%)	704 (77.6%)	
Yes	395 (24.4%)	203 (22.4%)	
**Hypertension Status**			0.156
No	833 (51.4%)	493 (54.3%)	
Yes	789 (48.6%)	415 (45.7%)	
**Nephrotoxic Medication Use**			0.3771
No	751 (46.3%)	437 (48.1%)	
Yes	871 (53.7%)	471 (51.9%)	
**Has Depression**			0.485
No	1096 (72.5%)	609 (71.1%)	
Yes	416 (27.5%)	247 (28.9%)	
**CESD**			0.784
Mean (SD)	11.5 (10.9)	11.4 (10.9)	
Median [Min, Max]	8.00 [0, 54.0]	8.00 [0, 52.0]	
**Current Smoking Status**			0.214
Currently smoking	428 (28.0%)	263 (30.4%)	
Not currently smoking	1100 (72.0%)	602 (69.6%)	
**Hazardous Drinking Since Last Visit**			0.002
No	940 (91.7%)	502 (86.9%)	
Yes	85 (8.3%)	76 (13.1%)	
**Binge Drinking Since Last Visit**			0.157
No	1222 (75.3%)	653 (71.9%)	
Yes	246 (15.2%)	153 (16.9%)	
Missing	154 (9.5%)	102 (11.2%)	
**Used Marijuana Since Last Visit**			0.004
No	1067 (65.8%)	550 (60.6%)	
Yes	460 (28.4%)	314 (34.6%)	
Missing	95 (5.9%)	44 (4.8%)	
**Cocaine Use Since Last Visit**			0.018
No	1405 (86.6%)	766 (84.4%)	
Yes	120 (7.4%)	96 (10.6%)	
Missing	97 (6.0%)	46 (5.1%)	
**Heroin Use Since Last Visit**			<0.001
No	1503 (92.7%)	827 (91.1%)	
Yes	21 (1.3%)	36 (4.0%)	
Missing	98 (6.0%)	45 (5.0%)	
**Other Drug Use Since Last Visit**			0.04
No	1354 (83.5%)	740 (81.5%)	
Yes	171 (10.5%)	124 (13.7%)	
Missing	97 (6.0%)	44 (4.8%)	
**Injection Drug Use Since Last Visit**			0.373
No	1511 (93.2%)	853 (93.9%)	
Yes	16 (1.0%)	12 (1.3%)	
Missing	95 (5.9%)	43 (4.7%)	

**Table 2:** Prevalence of Chronic Kidney Disease by Participant Characteristics, Overall and Stratified by HIV Status

		Overall		PLWH		PLWoH	
		N	Prevalence (%)	N	Prevalence (%)	N	Prevalence (%)
**HIV serostatus at time of interview**	HIV seronegative	88/908	9.7	-	-	-	-
	HIV positive	294/1622	18.1	-	-	-	-
**Sex at Birth**	Female	285/1712	16.7	224/1190	18.8	61/522	11.7
	Male	97/818	11.9	70/432	16.2	27/386	7.0
**Race/Ethnicity**	Non-Hispanic White	70/572	12.2	51/297	17.2	19/275	6.9
	Non-Hispanic Black	260/1561	16.7	201/1070	18.8	59/491	12.0
	Non-Hispanic Other	17/94	18.1	15/62	24.2	2/32	6.3
	Hispanic	35/303	11.6	27/193	14.0	8/110	7.3
**Education**	Less than high school	92/623	14.8	67/428	15.7	25/195	12.8
	Completed high school	101/642	15.7	82/452	18.1	19/190	10.0
	Some college	118/666	17.7	93/438	21.2	25/228	11.0
	Completed four-year college degree	40/282	14.2	30/162	18.5	10/120	8.3
	Attended/Completed graduate school	31/315	9.8	22/142	15.5	9/173	5.2
**Marital Status**	Legally/Common-law Married	73/545	13.4	49/322	15.2	24/223	10.8
	Not Married but Living with Partner	24/218	11.0	18/116	15.5	6/102	5.9
	Widowed	50/179	27.9	41/134	30.6	9/45	20.0
	Divorced	50/291	17.2	42/201	20.9	8/90	8.9
	Separated	14/137	10.2	7/83	8.4	7/54	13.0
	Never Married	129/947	13.6	102/626	16.3	27/321	8.4
	Other	33/155	21.3	27/104	26.0	6/51	11.8
**Employment Status**	Not Employed	55/438	12.6	43/268	16.0	12/170	7.1
	Employed Full-Time	57/716	8.0	45/442	10.2	12/274	4.4
	Employed Part-Time	26/242	10.7	21/156	13.5	5/86	5.8
	Retired	71/309	23.0	47/149	31.5	24/160	15.0
	Student	0/15	0.0	0/9	0.0	0/6	0.0
	Disability	163/749	21.8	129/560	23.0	34/189	18.0
**Income**	$6000 or less	26/182	14.3	16/109	14.7	10/73	13.7
	$6001–$12,000	136/643	21.2	106/470	22.6	30/173	17.3
	$12,001–$18,000	60/310	19.4	50/214	23.4	10/96	10.4
	$18,001–$24,000	30/198	15.2	25/139	18.0	5/59	8.5
	$24,001–$30,000	23/166	13.9	19/110	17.3	4/56	7.1
	$30,001–$36,000	15/140	10.7	12/85	14.1	3/55	5.5
	$36,001–$75,000	44/394	11.2	29/233	12.5	15/161	9.3
	$75,001–$100,000	7/112	6.3	5/57	8.8	2/55	3.6
	$100,001–$150,000	10/126	7.9	10/64	15.6	0/62	0.0
	$150,001–$200,000	7/56	12.5	4/30	13.3	3/26	11.5
	More than $200,000	2/58	3.5	1/24	4.2	1/34	2.9
**Has Diabetes**	No	240/1928	12.5	190/1224	15.5	50/704	7.1
	Yes	142/598	23.8	104/395	26.3	38/203	18.7
**Hypertension Status**	No	126/1326	9.5	107/833	12.9	19/493	3.9
	Yes	256/1204	21.3	187/789	23.7	69/415	16.6
**Has Depression**	No	261/1705	15.3	200/1096	18.3	61/609	10.0
	Yes	92/663	13.9	71/416	17.1	21/247	8.5
**Nephrotoxic Medication Use**	No	124/1188	10.4	99/751	13.2	25/437	5.7
	Yes	258/1342	19.2	195/871	22.4	63/471	13.4
**Current Smoking Status**	Not currently smoking	248/1702	14.6	198/1100	18.0	50/602	8.3
	Currently smoking	105/691	15.2	72/428	16.8	33/263	12.6
**Binge Drinking Since Last Visit**	No	294/1875	15.7	229/1222	18.7	65/653	10.0
	Yes	38/399	9.5	29/246	11.8	9/153	5.9
**Used Marijuana Since Last Visit**	No	261/1617	16.1	206/1067	19.3	55/550	10.0
	Yes	92/774	11.9	64/460	13.9	28/314	8.9
**Cocaine Use Since Last Visit**	No	315/2171	14.5	247/1405	17.6	68/766	8.9
	Yes	36/216	16.7	22/120	18.3	14/96	14.6
**Heroin Use Since Last Visit**	No	343/2330	14.7	266/1503	17.7	77/827	9.3
	Yes	9/57	15.8	4/21	19.1	5/36	13.9
**Other Drug Use Since Last Visit**	No	323/2094	15.4	247/1354	18.2	76/740	10.3
	Yes	29/295	9.8	22/171	12.9	7/124	5.7
**Injection Drug Use Since Last Visit**	No	349/2364	14.8	268/1511	17.7	81/853	9.5
	Yes	4/28	14.3	2/16	12.5	2/12	16.7

**Table 3:** Prevalence of Nephrotoxic Medication Use by Visit and HIV Status

Visit Number	HIV Status	Total Participants	Participants Using Nephrotoxic Medications (n)	Nephrotoxic Medication Use (%)
101	Overall	2,530	1,342	53.0
101	HIV-	908	471	51.9
101	HIV+	1,622	871	53.7
102	Overall	2,985	1,624	54.4
102	HIV-	1,074	566	52.7
102	HIV+	1,911	1,058	55.4
103	Overall	1,351	699	51.7
103	HIV-	440	213	48.4
103	HIV+	911	486	53.4

* Nephrotoxic medication use was defined based on expert review of medications with established nephrotoxic properties (see Supplement Table 1).

**Table 4.** GEE Analysis Results: Factors Associated with Chronic Kidney Disease

Variable	RR	95% CI	P-value
HIV Serostatus (HIV Positive vs. HIV Negative)	**1.37**	**(1.48, 1.28)**	**<.0001**
Age (per unit increase)	**1.03**	**(1.03, 1.03)**	**<.0001**
Sex (Female vs. Male)	**1.16**	**(1.23, 1.08)**	**<.0001**
Race: Non-Hispanic Black (vs. Non-Hispanic White)	**1.19**	**(1.11, 1.27)**	**<.0001**
Race: Hispanic (vs. Non-Hispanic White)	**2.03**	**(1.86, 2.21)**	**<.0001**
Race: Non-Hispanic Other (vs. Non-Hispanic White)	**2.64**	**(2.31, 3.02)**	**<.0001**
Education: Completed high school (HS) vs. < HS	0.83	(0.68, 1.03)	0.087
Education: Some college vs. < HS	0.87	(0.72, 1.07)	0.184
Education: Completed/graduate college vs. < HS	0.81	(0.64, 1.02)	0.069
Education: Attended/completed post-grad vs. < HS	0.86	(0.69, 1.06)	0.163
Marital: Not Married but Living with Partner (vs. Married)	1.16	(1.01, 1.32)	**0.0364**
Marital: Widowed (vs. Married)	1.16	(0.97, 1.4)	0.1059
Marital: Divorced (vs. Married)	1.07	(0.93, 1.25)	0.3432
Marital: Separated (vs. Married)	1.16	(1.0, 1.36)	0.0556
Marital: Never Married (vs. Married)	1.01	(0.9, 1.12)	0.8762
Marital: Other (vs. Married)	1.02	(0.89, 1.18)	0.7433
Income (per unit increase)	0.99	(0.98, 1.0)	**0.005**
Diabetes (Yes vs. No)	1.26	(1.17, 1.35)	**<.0001**
Hypertension (Yes vs. No)	1.06	(0.97, 1.15)	0.2043
Depression (Yes vs. No)	1.04	(0.96, 1.14)	0.3355
Nephrotoxic Medication (Yes vs. No)	1.04	(0.96, 1.14)	0.3355
Current Smoker (vs. Non-Smoker)	1.09	(1.31, 0.91)	0.3352
Marijuana Use (Yes vs. No)	1.1	(0.96, 1.25)	0.1655
Cocaine Use (Yes vs. No)	0.92	(0.75, 1.12)	0.3918
Heroin Use (Yes vs. No)	0.69	(0.28, 1.71)	0.4257
Other Drug Use (Yes vs. No)	0.96	(0.76, 1.21)	0.7075
Injection Drug Use (Yes vs. No)	1	(0.97, 1.03)	0.9276
BMI (per unit increase)	1.02	(0.99, 1.05)	0.2426

## Data Availability

MWCCS data are available to qualified researchers through the MWCCS Data Analysis and Coordination Center (https://mwccs.org/data-access).

## References

[1] Diana NE, Naicker S. Update on current management of chronic kidney disease in patients with HIV infection. Int J Nephrol Renovasc Dis. 2016;9:223–34. Epub 2016/10/04. doi: 10.2147/ijnrd.S93887. 27695357 PMC5033612

[2] Rivera FB, Ansay MFM, Golbin JM, Alfonso PGI, Mangubat GFE, Menghrajani RHS, HIV-Associated Nephropathy in 2022. Glomerular Dis. 2023;3(1):1–11. Epub 20221024. doi: 10.1159/000526868. 36816427 PMC9936764

[3] Jotwani V, Li Y, Grunfeld C, Choi AI, Shlipak MG. Risk factors for ESRD in HIV-infected individuals: traditional and HIV-related factors. Am J Kidney Dis. 2012;59(5):628–35. Epub 2011/12/31. doi: 10.1053/j.ajkd.2011.10.050. 22206742 PMC3324595

[4] Bickel M, Marben W, Betz C, Khaykin P, Stephan C, Gute P, End-stage renal disease and dialysis in HIV-positive patients: observations from a long-term cohort study with a follow-up of 22 years. HIV Med. 2013;14(3):127–35. Epub 2012/09/22. doi: 10.1111/j.1468-1293.2012.01045.x. 22994610

[5] Wyatt CM, Klotman PE, D’Agati VD. HIV-associated nephropathy: clinical presentation, pathology, and epidemiology in the era of antiretroviral therapy. Semin Nephrol. 2008;28(6):513–22. doi: 10.1016/j.semnephrol.2008.08.005. 19013322 PMC2656916

[6] Ahmed GY, Saha C, Almusalami EM, Rabaan AA, Alhumaid S, Ali AA, Prevalence of Depression in Elderly People Living With HIV: A Systematic Review and Meta-analysis. Infectious Microbes & Diseases. 2023;5(4):167–71. doi: 10.1097/im9.0000000000000132.

[7] Zhang Z, He P, Liu M, Zhou C, Liu C, Li H, Association of Depressive Symptoms with Rapid Kidney Function Decline in Adults with Normal Kidney Function. Clin J Am Soc Nephrol. 2021;16(6):889–97. Epub 2021/05/31. doi: 10.2215/cjn.18441120. 34052796 PMC8216614

[8] Meng F, Qi Y, Chen X, Yan X, Huang H, He F. The synergistic effect of depression and moderate chronic kidney disease on the all-cause and cardiovascular disease mortality among adults: a retrospective cohort study. BMC Nephrol. 2022;23(1):330. Epub 2022/10/12. doi: 10.1186/s12882-022-02957-7. 36221061 PMC9554995

[9] Kurani S, Jeffery MM, Thorsteinsdottir B, Hickson LJ, Barreto EF, Haag J, Use of Potentially Nephrotoxic Medications by U.S. Adults with Chronic Kidney Disease: NHANES, 2011–2016. J Gen Intern Med. 2020;35(4):1092–101. Epub 2019/12/04. doi: 10.1007/s11606-019-05557-8. months, Dr. McCoy was also supported by the AARP^®^ Quality Measure Innovation Grant through a collaboration with OptumLabs^®^ and the NQF Measure Incubator. Dr. Jeffery has received research support through Mayo Clinic from the National Heart, Lung and Blood Institute (R56HL130496 and R21HL140287), the Agency for Healthcare Research and Quality (R01HS025164), the American Cancer Society (131611-RSGI-17–154-01-CPHPS), the Food and Drug Administration-funded Yale-Mayo CERSI (U01FD 05938), and the National Center for Advancing Translational Sciences (UL1TR 02377; U01TR 02743). In the past 36 months, Dr. Shah has received research support through Mayo Clinic from the Food and Drug Administration to establish Yale-Mayo Clinic Center for Excellence in Regulatory Science and Innovation (CERSI) program (U01FD005938); the Centers of Medicare and Medicaid Innovation under the Transforming Clinical Practice Initiative (TCPI); the Agency for Healthcare Research and Quality (R01HS025164; R01HS025402; R03HS025517); the National Heart, Lung and Blood Institute of the National Institutes of Health (NIH) (R56HL130496; R01HL131535); the National Science Foundation; and the Patient Centered Outcomes Research Institute (PCORI) to develop a Clinical Data Research Network (LHSNet). 31792867 PMC7174522

[10] Papatriantafyllou E, Efthymiou D, Markopoulou M, Sakellariou E-M, Vassilopoulou E. The Effects of Use of Long-Term Second-Generation Antipsychotics on Liver and Kidney Function: A Prospective Study. Diseases. 2022;10(3):48. 35892742 10.3390/diseases10030048 PMC9332711

[11] Damba JJ, Bodenstein K, Lavin P, Drury J, Sekhon H, Renoux C, Psychotropic drugs and adverse kidney effects: a systematic review of the past decade of research. CNS drugs. 2022;36(10):1049–77. 36161425 10.1007/s40263-022-00952-y

[12] Capuron L, Gumnick JF, Musselman DL, Lawson DH, Reemsnyder A, Nemeroff CB, Neurobehavioral effects of interferon-alpha in cancer patients: phenomenology and paroxetine responsiveness of symptom dimensions. Neuropsychopharmacology. 2002;26(5):643–52. Epub 2002/04/03. doi: 10.1016/s0893-133x(01)00407-9. 11927189

[13] D’Souza G, Bhondoekhan F, Benning L, Margolick JB, Adedimeji AA, Adimora AA, Characteristics of the MACS/WIHS Combined Cohort Study: Opportunities for Research on Aging With HIV in the Longest US Observational Study of HIV. Am J Epidemiol. 2021;190(8):1457–75. Epub 2021/03/07. doi: 10.1093/aje/kwab050. 33675224 PMC8484936

[14] Levin A, Stevens PE, Bilous RW, Coresh J, De Francisco AL, De Jong PE, Kidney Disease: Improving Global Outcomes (KDIGO) CKD Work Group. KDIGO 2012 clinical practice guideline for the evaluation and management of chronic kidney disease. Kidney international supplements. 2013;3(1):1–150.

[15] Radloff LS. The CES-D Scale: A self-report depression scale for research in the general population. Applied Psychological Measurement. 1977;1(3):385–401. doi: 10.1177/014662167700100306.

[16] Cook JA, Cohen MH, Burke J, Grey D, Anastos K, Kirstein L, Effects of depressive symptoms and mental health quality of life on use of highly active antiretroviral therapy among HIV-seropositive women. JAIDS Journal of Acquired Immune Deficiency Syndromes. 2002;30(4):401–9. 12138346 10.1097/00042560-200208010-00005

[17] Armstrong NM, Surkan PJ, Treisman GJ, Sacktor NC, Irwin MR, Teplin LA, Optimal metrics for identifying long term patterns of depression in older HIV-infected and HIV-uninfected men who have sex with men. Aging Ment Health. 2019;23(4):507–14. Epub 2018/02/10. doi: 10.1080/13607863.2017.1423037. 29424569 PMC6085148

[18] John Preston PDaPAD. Quick Reference To Psychiatric Medications. 2023.

[19] Stottlemyer BA, Abebe KZ, Palevsky PM, Fried L, Schulman IH, Parikh CR, Expert Consensus on the Nephrotoxic Potential of 195 Medications in the Non-intensive Care Setting: A Modified Delphi Method. Drug Saf. 2023;46(7):677–87. Epub 2023/05/24. doi: 10.1007/s40264-023-01312-5. to the research, authorship, and/or publication of this article: KZA has received support from AKI grant U01DK130010. LF has served as a consultant for Bayer and is a member of the DSMB for Novo Nordisk and CSL Behring. ES serves on the Editorial Board of CJASN and has received royalties as an author for UpToDate. FPW has received support from AKI grants R01DK113191, P30DK079310, and R01HS027626. MRW has served as a scientific advisor for Bayer, AstraZeneca, Boehringer-Ingelheim, Janssen, Merck, and Novo Nordisk. SKG has received support from AKI grants U01DK130010 and R01DK121730–01. Authors BAS, PMP, IHS, CRP, EP, OMG, EH declare that they have no conflicts of interest. 37223847 PMC10208182

[20] Westreich D, Greenland S. The table 2 fallacy: presenting and interpreting confounder and modifier coefficients. American journal of epidemiology. 2013;177(4):292–8. 23371353 10.1093/aje/kws412 PMC3626058

[21] Naicker S, Rahmanian S, Kopp JB. HIV and chronic kidney disease. Clin Nephrol. 2015;83(7 Suppl 1):32–8. doi: 10.5414/cnp83s032. 25725239 PMC4536633

[22] Sarfo FS, Keegan R, Appiah L, Shakoor S, Phillips R, Norman B, High prevalence of renal dysfunction and association with risk of death amongst HIV-infected Ghanaians. Journal of Infection. 2013;67(1):43–50. 23542785 10.1016/j.jinf.2013.03.008

[23] Ryom L, Lundgren JD, Law M, Kirk O, El-Sadr W, Bonnet F, Serious clinical events in HIV-positive persons with chronic kidney disease. Aids. 2019;33(14):2173–88. 31385862 10.1097/QAD.0000000000002331

[24] Matías-García PR, Ward-Caviness CK, Raffield LM, Gao X, Zhang Y, Wilson R, DNAm-based signatures of accelerated aging and mortality in blood are associated with low renal function. Clinical epigenetics. 2021;13(1):121. 34078457 10.1186/s13148-021-01082-w PMC8170969

[25] Legrand K, Speyer E, Stengel B, Frimat L, Sime WN, Massy ZA, Perceived health and quality of life in patients with CKD, including those with kidney failure: findings from national surveys in France. American Journal of Kidney Diseases. 2020;75(6):868–78. 31879215 10.1053/j.ajkd.2019.08.026

[26] Cohen SD, Kopp JB, Kimmel PL. Kidney diseases associated with human immunodeficiency virus infection. New England Journal of Medicine. 2017;377(24):2363–74. 29236630 10.1056/NEJMra1508467

[27] Hara M, Suganuma A, Yanagisawa N, Imamura A, Hishima T, Ando M. Atazanavir nephrotoxicity. Clinical kidney journal. 2015;8(2):137–42. 25815168 10.1093/ckj/sfv015 PMC4370314

[28] Winston JA. HIV and CKD epidemiology. Advances in chronic kidney disease. 2010;17(1):19–25. 20005485 10.1053/j.ackd.2009.08.006

[29] Nanni MG, Caruso R, Mitchell AJ, Meggiolaro E, Grassi L. Depression in HIV infected patients: a review. Current psychiatry reports. 2015;17:1–11. 25413636 10.1007/s11920-014-0530-4

[30] Atlanta GUDoHaHS, Centers for Disease Control and Prevention. Chronic Kidney Disease in the United States, 2023. CDC. 2023.

[31] Tsai YC, Chiu YW, Hung CC, Hwang SJ, Tsai JC, Wang SL, Association of symptoms of depression with progression of CKD. Am J Kidney Dis. 2012;60(1):54–61. Epub 2012/04/13. doi: 10.1053/j.ajkd.2012.02.325. 22495469

[32] Lustman PJ, Clouse RE, Griffith LS, Carney RM, Freedland KE. Screening for depression in diabetes using the Beck Depression Inventory. Psychosom Med. 1997;59(1):24–31. Epub 1997/01/01. doi: 10.1097/00006842-199701000-00004. 9021863

[33] Guo Y, Musselman DL, Manatunga AK, Gilles N, Lawson KC, Porter MR, The diagnosis of major depression in patients with cancer: a comparative approach. Psychosomatics. 2006;47(5):376–84. Epub 2006/09/09. doi: 10.1176/appi.psy.47.5.376. 16959925

[34] Abraham AG, Althoff KN, Jing Y, Estrella MM, Kitahata MM, Wester CW, End-stage renal disease among HIV-infected adults in North America. Clin Infect Dis. 2015;60(6):941–9. Epub 2014/11/20. doi: 10.1093/cid/ciu919. 25409471 PMC4357817

[35] Ong LT, Chee NMZ, Loh AJC. Risk of renal impairment in atypical antipsychotics: a systematic review and meta-analysis. European Journal of Clinical Pharmacology. 2024;80(10):1435–44. 38916726 10.1007/s00228-024-03714-5

[36] Bendz H, Aurell M, Balldin J, Mathe A, Sjödin I. Kidney damage in long-term lithium patients: a cross-sectional study of patients with 15 years or more on lithium. Nephrology Dialysis Transplantation. 1994;9(9):1250–4. 7816284

[37] Presne C, Fakhouri F, Noël L-H, Stengel B, Even C, Kreis H, Lithium-induced nephropathy: rate of progression and prognostic factors. Kidney international. 2003;64(2):585–92. 12846754 10.1046/j.1523-1755.2003.00096.x

[38] Potok OA, Katz R, Bansal N, Siscovick DS, Odden MC, Ix JH, The Difference Between Cystatin C- and Creatinine-Based Estimated GFR and Incident Frailty: An Analysis of the Cardiovascular Health Study (CHS). Am J Kidney Dis. 2020;76(6):896–8. Epub 2020/07/20. doi: 10.1053/j.ajkd.2020.05.018. 32682698 PMC7967899

